# Cell Type-Specific Gene Network-Based Analysis Depicts the Heterogeneity of Autism Spectrum Disorder

**DOI:** 10.3389/fncel.2020.00059

**Published:** 2020-03-19

**Authors:** Jinting Guan, Yiping Lin, Guoli Ji

**Affiliations:** ^1^Department of Automation, Xiamen University, Xiamen, China; ^2^National Institute for Data Science in Health and Medicine, Xiamen University, Xiamen, China; ^3^Innovation Center for Cell Signaling Network, Xiamen University, Xiamen, China

**Keywords:** cell type-specific, gene network, cell type heterogeneity, autism, gene function

## Abstract

Autism spectrum disorder (ASD) is a complex neuropsychiatric disorder characterized by substantial heterogeneity. To identify the convergence of disease pathology on common pathways, it is essential to understand the correlations among ASD candidate genes and study shared molecular pathways between them. Investigating functional interactions between ASD candidate genes in different cell types of normal human brains may shed new light on the genetic heterogeneity of ASD. Here we apply cell type-specific gene network-based analysis to analyze human brain nucleus gene expression data and identify cell type-specific ASD-associated gene modules. ASD-associated modules specific to different cell types are relevant to different gene functions, for instance, the astrocytes-specific module is involved in functions of axon and neuron projection guidance, GABAergic interneuron-specific modules are involved in functions of postsynaptic membrane, extracellular matrix structural constituent, and ion transmembrane transporter activity. Our findings can promote the study of cell type heterogeneity of ASD, providing new insights into the pathogenesis of ASD. Our method has been shown to be effective in discovering cell type-specific disease-associated gene expression patterns and can be applied to other complex diseases.

## Introduction

Autism spectrum disorder (ASD) is a complex neuropsychiatric disorder with substantial phenotypic and genetic heterogeneity, characterized by impairments in social interaction and communication, and repetitive and restricted behaviors. Until now, about a thousand genes, each with different functions, have been linked to ASD, while it remains unclear how disruptions in these genes can lead to a common clinical phenotype. For identifying potential converged molecular pathways, it is essential to study the correlations among ASD candidate genes. Since ASD is believed to result from functional aberrations within brains, it was hypothesized that investigating functional relationships between ASD candidate genes in normal human brains may provide convergent mechanistic insight into the genetic heterogeneity of ASD (Mahfouz et al., 2015). With this hypothesis, several studies constructed a gene co-expression network based on bulk transcriptomic data of normal human brains and mapped ASD candidate genes or/and cell type markers to the gene modules, whose functions were analyzed for identifying the pathways and cell types which may be dysregulated in ASD (Ben-David and Shifman, 2012; Parikshak et al., 2013; Willsey et al., 2013; Mahfouz et al., 2015).

The human brain is a highly heterogeneous organ with different sets of cell types that are highly interconnected. Genes may demonstrate diverse functions across different brain cell types. Although bulk transcriptomic studies revealed convergence of disease pathology on common pathways (Voineagu et al., 2011; Gupta et al., 2014), the human brain cell type-specific molecular pathology of ASD is still needed to study. Here we hypothesize further that different gene functions in different human brain cell types may be dysregulated in ASD; investigating functional interactions between ASD candidate genes in different cell types of normal human brains may provide new insight into the genetic heterogeneity of ASD. Therefore, it is essential to construct gene networks in a cell type-specific way for identifying the vulnerable cell types and converged pathways among ASD candidate genes in different cells.

In this paper, we apply cell type-specific gene network-based analysis to analyze human brain nucleus gene expression data. To study the heterogeneity of ASD in aberrant gene expression between cell types, we identify cell type-specific ASD-associated gene modules and analyze dysregulated functions in ASD specific to cell types. Compared with other studies, our method has been shown to be effective in discovering cell type-specific disease-associated gene expression patterns.

## Materials and Methods

### Gene Expression Data

We used the gene expression data of 15,928 human brain nuclei from middle temporal gyrus of human cortex (Hodge et al., 2019). These nuclei were from eight human donor brains, of which 15,206 were from postmortem donors with no known neuropsychiatric or neurological conditions and 722 were from distal and normal tissues of neurosurgical donors. We downloaded the data from Allen Institute for Brain Science and preprocessed it with R package of scran (Lun et al., 2016), including the quality control of nuclei and genes, removing a minority of nuclei assigned to different cell cycle phases and normalizing the data. We used ComBat to regress out the technical factor (seq_batch) which contributed to the heterogeneity of gene expression. Nuclear and mitochondrial genes downloaded from Human MitoCarta2.0 (Calvo et al., 2016) were excluded and protein-coding genes were retained. Then we obtained the expression level of 17,120 protein-coding genes in 12,506 nuclei, including 8,994, 2,762, 227, 3, 15, 112, 133, and 260 nuclei from glutamatergic neuron, GABAergic interneuron, astrocytes, endothelial, microglia, oligodendrocytes, oligodendrocyte precursor cell (OPC), and unclassified cell class (denoted by “No”), respectively. The classified nuclei were also defined into 75 distinct cell clusters, including 24, 45, 2, 1, 1, 1, and 1 cell clusters from glutamatergic neuron, GABAergic interneuron, astrocytes, endothelial, microglia, oligodendrocytes, and OPC. Then we used scran to obtain 7,011 highly variable protein-coding genes across all nuclei for performing subsequent analyses, which were genes with biological components that are significantly greater than zero at a false discovery rate (FDR) of 0.1.

### Construction of Cell Type-Related Gene Network

For each cell type, we extracted the gene expression data of nuclei from the cell type and constructed a gene co-expression network using WGCNA (weighted gene co-expression network analysis) (Langfelder and Horvath, 2008). The unsigned gene network was built using function of *blockwiseModules* with parameters of *corType* = “pearson,” *TOMType* = “signed,” *minModuleSize* = 30, *minKMEtoStay* = 0.2, and *mergeCutHeight* = 0.2. To assess the preservation between gene modules built from each cell type and from other cell types, the function of *modulePreservation* (Langfelder et al., 2011) in WGCNA was applied to calculate module preservation statistics. The median of Zsummary values was used to further evaluate if a candidate gene module is cell type-specific. We calculated the correlation of module eigengene (the first principal component) and each gene, defined as module membership. For the genes with top 50 module membership, we applied Cytoscape (Shannon et al., 2003) to plot the correlation network based on the topology overlap matrix (TOM) from WGCNA.

### Curated ASD Candidate Gene Set

A total of 822 ASD candidate genes were downloaded from the gene scoring module in Simons Foundation Autism Research Initiative (SFARI), which include the genes from categories of S (syndromic), 1 (high confidence), 2 (strong candidate), 3 (suggestive evidence), and 4 (minimal evidence).

### Calculation of Cell Type Specificity of Genes

To identify cell type-specific gene modules, we first calculated cell type specificity of genes using a method similar to that in the study of (McKenzie et al., 2018), where specificity was defined as the minimum fold change in expression between the cell type of interest and each of the other cell types. For this, we calculated the counts per million (CPM) using the R package of edgeR (Robinson et al., 2010). The specificity of gene *g* in the interested cell type indexed by *c* was calculated as:

$$\begin{array}{l} specificity_{g,c}=\underset{r\epsilon \left\{1,2,\ldots ,k\right\}\backslash c}{\min}\frac{\sum_{i=1}^{N_{c}}\text{exp}\left(i,g,c\right)/N_{c}}{\sum_{j=1}^{N_{r}}\text{exp}\left(j,g,r\right)/N_{r}} \end{array}$$

where each of *k* cell types was denoted by a numerical index from the set{1,2….,*k*}, *r* denoted one cell type from the reference cell set, *N*_*c*_ and *N*_*r*_ were the numbers of nuclei classified into cell types of *c* and *r* respectively, exp(*i,g,c*) denotes the expression of gene *g* in nucleus *i* from cell type of *c*. The genes with top 500 specificity values were used as cell type-specific genes to assess the cell type enrichment for each considered gene module. The gene modules built from a cell type significantly enriched with the specific genes of the cell type but not enriched with other kinds of cell type-specific genes are considered as candidate cell type-specific gene modules.

### Gene Set Enrichment and Functional Annotation Analyses

For a considered gene module, we used hypergeometric tests to assess the significance of enrichment of cell type-specific genes or ASD candidate genes. The correction for multiple testing was performed by controlling FDR with the Benjamini–Hochberg method. Gene ontology analysis was performed using the R package of clusterProfiler (Yu et al., 2012), with background genes set as the genes in the analyzed expression matrix. The GO term whose FDR-adjusted *P*-value <0.1 and the number of genes in the term is not less than five was reported. The function of *simplify* was used to remove redundant significant GO terms with default parameters.

## Results

### Overview of Analysis Workflow

**Figure 1:**
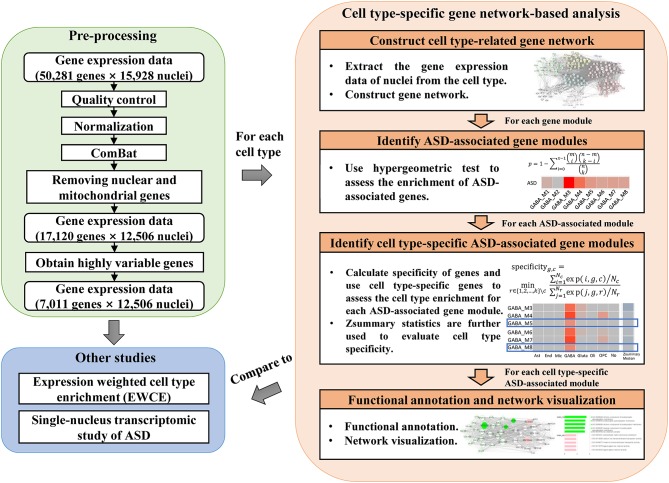
The analysis workflow which includes pre-processing of gene expression data, cell type-specific gene network-based analysis, and comparison to other studies.

To study the dysregulated functions in ASD specific to cell types, we applied cell type-specific gene network-based analysis to identify cell type-specific ASD-associated gene modules. The analysis workflow can be seen in Figure 1. After pre-processing the data matrix (see Materials and Methods), we first used the broad definition of cell type, the classification of cell classes, to identify cell class-specific ASD-associated gene modules. Specifically, for each cell class, we constructed a gene co-expression network (Supplementary Table 1). Then SFARI ASD candidate genes were used to identify ASD-associated gene modules. The gene modules significantly enriched with ASD candidate genes (FDR-corrected *P*-value <0.1) are considered as ASD-associated gene modules. Next, we calculated cell class specificity of genes (see Materials and Methods), and cell class-specific genes (Supplementary Table 2) were used to assess the cell class enrichment for each ASD-associated module. The ASD-associated gene modules built from a cell class significantly enriched with the specific genes of the cell class (FDR-corrected *P*-value <0.1) but not enriched with other kinds of cell class-specific genes are considered as candidate cell class-specific ASD-associated gene modules. Then the function of *modulePreservation* in WGCNA was applied to calculate module preservation statistics between gene modules build from each cell class and from other cell classes (Supplementary Table 1). As Zsummary <2 implies no evidence for module preservation (Langfelder et al., 2011), we reported the candidate cell class-specific ASD-associated gene modules whose medians of Zsummary are smaller than two as cell class-specific ASD-associated gene modules. These gene modules are not preserved from the considered cell class to other cell classes, which implies they are cell type-specific. In addition, we also adopted the classification of cell clusters and identified cell cluster-specific ASD-associated gene modules. The classification of cell clusters and the calculated cell cluster specificity of genes can be seen in Supplementary Table 3. By applying functional annotation analysis for cell type-specific ASD-associated gene modules, we study the probably affected functions by ASD specific to cell types. Network visualization was performed to prioritize genes that may be used as therapeutic targets of ASD. Lastly, we applied expression weighted cell type enrichment (EWCE) (Skene and Grant, 2016) and also analyzed the single-nucleus gene expression data of cortical tissue samples from ASD patients and healthy controls reported in (Velmeshev et al., 2019) to validate our results.

### Cell Type-Specific Gene Network-Based Analysis

**Figure 2:**
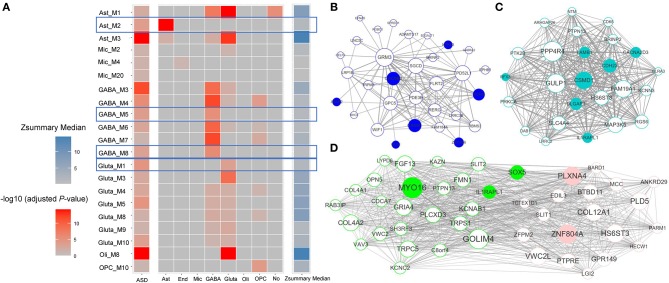
**(A)** The enrichment of cell class-specific genes for ASD-associated gene modules along with medians of Zsummary. Each grid in the first column demonstrates the significance of enrichment of ASD candidate genes; each grid in the last column demonstrates the median of Zsummary; each grid in the other columns demonstrates the significance of enrichment of cell class-specific genes for each ASD-associated gene module. The cell class-specific ASD-associated gene modules are marked, which are Ast_M2, GABA_M5, GABA_M8, and Gluta_M1. The correlation networks between genes with top 50 module membership in cell class-specific ASD-associated gene modules were shown for **(B)** astrocytes, **(C)** glutamatergic neuron, and **(D)** GABAergic interneuron. The node size is proportional to the module membership of gene and the edge width is proportional to the entry of TOM between genes. SFARI ASD candidate genes from categories of S and 1–4 are filled with corresponding colors.

To analyze the affected gene functions in each cell class, we first detected ASD-associated gene modules for each cell class. We identified seven, six, three, three, one and one ASD-associated modules enriched in GO categories for glutamatergic neuron, GABAergic interneuron, astrocytes, microglia, oligodendrocytes, and OPC (Figure 2A). The top five significant GO terms enriched in these ASD-associated gene modules are shown as Supplementary Figure 1. To further study the dysregulated functions specific to cell classes, we identified six candidate cell class-specific ASD-associated gene modules, which are Ast_M2, GABA_M5, GABA_M8, Gluta_M1, Gluta_M3, and OPC_M10. Combining the Zsummary statistics calculated by WGCNA (Supplementary Table 1), modules of Ast_M2, GABA_M5, GABA_M8, and Gluta_M1 were retained, which are considered as cell class-specific ASD-associated gene modules. To identify the functions specific to each cell class-specific ASD-associated gene module, we report the GO terms only enriched in one module. The top specific GO terms enriched in cell class-specific ASD-associated gene modules can be seen in Table 1.

**Table 1:** The identified functional cell class-specific ASD-associated gene modules and their enriched top three significant GO terms.

**Cell class**	**Module ID**	**No. of genes**	**No. of ASD genes (*P*-value)**	**No. of cell class-specific genes (*P*-value)**	**Median of Zsummary**	**GO term**
Ast	M2	123	20 (0.00012)	38 (6.22E-15)	1.67	GO: 0007411 axon guidance
						GO: 0097485 neuron projection guidance
						GO: 0002040 sprouting angiogenesis
GABA	M5	179	24 (0.00012)	30 (3.73E-06)	1.56	GO: 0098948 intrinsic component of postsynaptic specialization membrane
						GO: 0099634 postsynaptic specialization membrane
						GO: 0098936 intrinsic component of postsynaptic membrane
	M8	90	13 (0.00114)	20 (1.43E-06)	0.74	GO: 0005201 extracellular matrix structural constituent
						GO: 0015085 calcium ion transmembrane transporter activity
						GO:0015276 ligand-gated ion channel activity
Gluta	M1	345	39 (0.00010)	44 (0.00030)	1.83	GO: 0019902 phosphatase binding
						GO: 0051018 protein kinase A binding

*The numbers of ASD candidate genes, cell class-specific genes and total genes in the module are shown along with FDR-corrected hypergeometric test P-values. For each module, the median of Zsummary values assessing the module preservation between the considered cell class and other cells is also listed. Ast, GABA, and gluta are short for astrocytes, GABAergic interneuron, and glutamatergic neuron*.

For astrocytes, the cell class-specific ASD-associated gene module is relevant to the functions of axon and neuron projection guidance, and sprouting angiogenesis. Astrocytes perform a variety of tasks from axon guidance and synaptic support to the control of the blood brain barrier and blood flow (Blackburn et al., 2009). The dysfunctions of astrocytes in modulating the development of synapse and interplay with neurons have already been shown to be implicated with ASD (Allen and Eroglu, 2017; Russo et al., 2017). For GABAergic interneuron, the functions of cell class-specific ASD-associated gene modules are related to postsynaptic membrane, extracellular matrix structural constituent, and ion transmembrane transporter activity. As to glutamatergic neuron, functions of phosphatase binding and protein kinase A binding may be dysregulated in ASD. Until now, it has been known that many ASD-associated mutations and variations are involved in the functioning of synapses (Ebrahimi-Fakhari and Sahin, 2015; Gilbert and Man, 2017). Neurons communicate with one another at synapses using two types of signals, electrical and chemical signals. At an electrical synapse, ions flow directly between cells. At a chemical synapse, chemical signals, called as neurotransmitters, pass messages from the presynaptic neuron to the postsynaptic one. Glutamate and GABA (gamma-aminobutyric acid) are major excitatory and inhibitory neurotransmitters in brains respectively. Alterations of GABAergic neuron-associated extracellular matrix (Wang et al., 2018b), the glutamatergic neuron-associated protein kinase activity (Bemben et al., 2015) and the mutation or dysfunction of phosphatases (Gross, 2017) have been linked to ASD.

For the cell class-specific ASD-associated gene modules, we plotted the correlation networks between the genes with top 50 module membership values (Figures 2B–D). The genes with top module membership are informative for the networks, and may be used as potential therapeutic targets for ASD. For example, in modules GABA_M5 and GABA_M8 (Figure 2D), genes *MYO16* and *ZNF804A* with the largest module membership are of note. Indeed, more and more recent studies have shown the associations between them and ASD (Anitha et al., 2014; Liu et al., 2015; Zhang et al., 2019).

### Comparison With Other Studies

To prove the effectiveness of our cell type-specific gene network-based analysis, we detected vulnerable cell types in ASD with EWCE (Skene and Grant, 2016) (Supplementary Table 4) using SFARI ASD genes from categories of S and 1–4, and our specificity matrices. Using our cell type-specific gene network-based analysis, four candidate cell cluster-specific ASD-associated gene modules were identified (Supplementary Table 5), including Inh L1-4 *LAMP5 LCP2*, Inh L1 *SST NMBR*, Astro L1-2 *FGFR3 GFAP*, and OPC L1-6 *PDGFRA*. Inh L1-4 *LAMP5 LCP2* and Inh L1 *SST NMBR* are cell clusters of GABAergic interneuron. From the result of EWCE, GABAergic interneuron is the most vulnerable cell type in ASD [also seen in Wang et al., 2018a], and Inh L1-4 *LAMP5 LCP2* and Inh L1 *SST NMBR* are also significant cell clusters (adjusted *P* = 0.07 and 0.001).

In addition, we found evidence from a recent single-nucleus transcriptomic study of ASD (Velmeshev et al., 2019) to validate our results. SFARI ASD genes were most overrepresented in L2/3 and L4 excitatory neurons, *VIP* and *SST*–expressing interneurons. Gene ontology analysis of differentially expressed genes identified in a cell type–specific way demonstrated that chemical synaptic transmission, axon guidance, neuronal migration, and GABA signaling were top dysregulated pathways. Besides, to assess if our identified cell class-specific ASD-associated gene modules can reemerge in the snRNA-seq dataset of ASD and controls, we used the expression data of protein-coding genes in this dataset to construct a gene co-expression network for each cell class using WGCNA. The module eigengene was associated with disease status to identify ASD-associated gene modules. We found that ASD-associated module denoted by blue constructed from excitatory neurons significantly includes the genes in Gluta_M1, ASD-associated modules denoted by yellow and brown constructed from inhibitory interneurons significantly include the genes in GABA_M5 and GABA_M8 (Supplementary Figure 2). The enriched functions in the blue module include protein serine/threonine kinase activity and negative regulation of phosphatase activity, the brown module is enriched with the functions of synaptic and postsynaptic membrane, integral and intrinsic component of synaptic and postsynaptic membrane, and ion gated channel activity, the yellow module is enriched with the functions of response to metal ion and metal ion transmembrane transporter activity. The results based on ASD snRNA-seq data validate the findings of our cell type-specific gene network-based analysis.

## Discussion

To identify the convergence of ASD pathology on common pathways, it is essential to understand the correlations among ASD candidate genes and study their shared molecular pathways. With the hypothesis that investigating functional relationships between ASD candidate genes in normal human brains may provide convergent mechanistic insight into the genetic heterogeneity of ASD, gene co-expression network was built based on normal human brain transcriptomic data sequenced by bulk RNA sequencing (Ben-David and Shifman, 2012; Mahfouz et al., 2015). The gene modules enriched with ASD candidate genes were analyzed for identifying the pathways which may be dysregulated in ASD. To depict the cell type heterogeneity of ASD, here we hypothesize further that different functions in different cell types may be dysregulated in ASD; investigating functional interactions between ASD candidate genes in different cell types in normal human brains may provide new insight into the genetic heterogeneity of ASD.

By conducting cell type-specific gene network-based analysis using human brain nucleus gene expression data, we identified cell type-specific ASD-associated gene modules, which were analyzed for studying dysregulated functions in ASD specific to cell types. Almost all enriched top GO terms in these cell type-specific ASD-associated gene modules are relevant to the corresponding cell types and have been proven to be associated with ASD. We found that different functions may be dysregulated in different human brain cell types, for instance, axon and neuron projection guidance in astrocytes; functions of postsynaptic membrane, extracellular matrix structural constituent and ion transmembrane transporter activity in GABAergic interneuron; functions of phosphatase binding and protein kinase A binding in glutamatergic neuron. By performing network visualization, we prioritize genes that may be used as potential therapeutic targets for ASD. In addition to using the definition of cell classes, we also used the classification of cell clusters to identify cell cluster-specific ASD-associated gene modules. Our findings provide new insights into the heterogeneity of ASD between different human brain cell types. Comparing with EWCE and single-cell transcriptomic study of ASD, it has been shown that cell type-specific gene network-based analysis is effective and can be applied to other kinds of complex diseases for identifying potential cell type-specific dysregulated molecular pathways, especially when the single-cell transcriptomic data of diseased samples is not available.

## Data Availability Statement

The datasets generated for this study can be found in the Allen Institute for Brain Science (https://portal.brain-map.org/atlases-and-data/rnaseq#transcriptomics).

## Author Contributions

JG conceived and designed the study. JG, YL, and GJ conducted the analyses and wrote the manuscript.

### Conflict of Interest

The authors declare that the research was conducted in the absence of any commercial or financial relationships that could be construed as a potential conflict of interest.

## Supplementary Material

Supplementary Figure 1 The identified functional ASD-associated gene modules and their enriched top five significant GO terms for cell classes of **(A)** astrocytes, **(B)** microglia, **(C)** GABAergic interneuron, **(D)** glutamatergic neuron, **(E)** OPC, and **(F)** oligodendrocytes.

Supplementary Figure 2 The constructed gene co-expression modules using snRNA-seq data of ASD and controls for cell classes of **(A)** astrocytes, **(B)** excitatory neurons, and **(C)** inhibitory interneurons. The module eigengene was associated with disease status for showing the correlation coefficient and the FDR-corrected *P*-value. The constructed gene modules are also tested if they significantly include the genes in our identified cell class-specific ASD-associated gene modules (Ast_M2, Gluta_M1, GABA_M5, and GABA_M8). The FDR-corrected hypergeometric test *P*-values are shown.

Supplementary Table 1 The gene co-expression modules built from each cell class. The module preservation statistics between gene modules built from each cell class and from other cell classes are also shown.

Supplementary Table 2 The definition of cell classes and the calculated cell class specificity of genes.

Supplementary Table 3 The definition of cell clusters and the calculated cell cluster specificity of genes.

Supplementary Table 4 The result of EWCE for cell classes and clusters.

Supplementary Table 5 The identified functional candidate cell cluster-specific ASD-associated gene modules and their enriched top five significant GO terms. The number of ASD candidate genes, cell cluster-specific genes and total genes in the module are shown along with FDR-corrected hypergeometric test *P*-values. Inh L1-4 *LAMP5 LCP2*: *LAMP5|LCP2*-expressing GABAergic interneuron; Inh L1 *SST NMBR*: *SST|NMBR*-expressing GABAergic interneuron; Astro L1-2 *FGFR3 GFAP*: *GFAP*-expressing astrocyte; OPC L1-6 *PDGFRA*: *PDGFRA*-expressing oligodendrocyte precursor cell.
