# Supplementary material for: Cell Type-Specific Gene Network-Based Analysis Depicts the Heterogeneity of Autism Spectrum Disorder
Source: Front Cell Neurosci. 2020 Mar 19;14:59. doi: 10.3389/fncel.2020.00059 (PMC7096557; doi:10.3389/fncel.2020.00059)
Supplement: Supplementary Table 5 — The identified functional candidate cell cluster-specific ASD-associated gene modules and their enriched top five significant GO terms. The number of ASD candidate genes, cell cluster-specific genes and total genes in the module are shown along with FDR-corrected hypergeometric test P-values. Inh L1-4 LAMP5 LCP2: LAMP5|LCP2-expressing GABAergic interneuron; Inh L1 SST NMBR: SST|NMBR-expressing GABAergic interneuron; Astro L1-2 FGFR3 GFAP: GFAP-expressing astrocyte; OPC L1-6 PDGFRA: PDGFRA-expressing oligodendrocyte precursor cell. [file Table_5.DOCX]

**Supplementary Table 5**. The identified functional candidate cell cluster-specific ASD-associated gene modules and their enriched top five significant GO terms. The number of ASD candidate genes, cell cluster-specific genes and total genes in the module are shown along with FDR-corrected hypergeometric test *P*-values. Inh L1-4 *LAMP5 LCP2*: *LAMP5|LCP2*-expressing GABAergic interneuron; Inh L1 *SST NMBR*: *SST|NMBR*-expressing GABAergic interneuron; Astro L1-2 *FGFR3 GFAP*: *GFAP*-expressing astrocyte; OPC L1-6 *PDGFRA*: *PDGFRA*-expressing oligodendrocyte precursor cell.

| **Cell cluster** | **Module ID** | **No. of genes** | **No. of ASD genes**  **(*P*-value)** | **No. of cell cluster-specific genes**  **(*P*-value)** | **GO term** |
| --- | --- | --- | --- | --- | --- |
| Inh L1-4 *LAMP5 LCP2* | M6 | 87 | 12 (0.01056) | 13  (0.03151) | GO: 0050804 modulation of chemical synaptic transmission |
|  |  |  |  |  | GO: 0099177 regulation of trans-synaptic signaling |
| Inh L1 *SST NMBR* | M6 | 72 | 12 (0.01542) | 14  (0.00818) | GO: 0006643 membrane lipid metabolic process |
|  |  |  |  |  | GO: 0046467 membrane lipid biosynthetic process |
|  |  |  |  |  | GO: 0042578 phosphoric ester hydrolase activity |
| Astro L1-2 *FGFR3 GFAP* | M30 | 106 | 15 (0.00817) | 14  (0.06570) | GO: 0098889 intrinsic component of presynaptic membrane |
|  |  |  |  |  | GO: 0099056 integral component of presynaptic membrane |
|  |  |  |  |  | GO: 0042734 presynaptic membrane |
|  |  |  |  |  | GO: 0099240 intrinsic component of synaptic membrane |
| OPC L1-6 *PDGFRA* | M10 | 104 | 14 (0.02481) | 17  (0.02485) | GO: 0045211 postsynaptic membrane |
|  |  |  |  |  | GO: 0097060 synaptic membrane |
|  |  |  |  |  | GO: 0098978 glutamatergic synapse |
